# Safety Analysis of Motorcycle Crashes in Seoul Metropolitan Area, South Korea: An Application of Nonlinear Optimal Scaling Methods

**DOI:** 10.3390/ijerph15122702

**Published:** 2018-11-30

**Authors:** Younshik Chung, Tai-Jin Song

**Affiliations:** 1 Department of Urban Planning and Engineering, Yeungnam University, Gyeongsan 38541, Korea; tpgist@yu.ac.kr; 2 Department of National Transport Big Data, The Korea Transport Institute, Sejong 30147, Korea

**Keywords:** motorcycle crash, motorcyclist injury severity, optimal scaling, categorical principal components analysis, nonlinear canonical correlation analysis

## Abstract

This study identifies the critical factors that affect motorcycle crash severity based on Korean motorcycle crash data in 2009. Motorcyclists, the environment, roadways, other vehicles involved in the crashes, and traffic flow characteristics were used as variables for identifying critical factors. Multivariable statistical methods were used to analyze the data, including categorical principal components analysis (CatPCA) and nonlinear canonical correlation analysis (NLCCA). The results indicate that the following factors are the most critical in increasing motorcycle crash severity: age (motorcyclists in their teens and over fifty years old), motorcycle speed over 30 km/h, speed over 50 km/h for other vehicles involved in the crash, crashes with heavy vehicles such as buses and trucks, crashes on roadways less than six meters wide, crashes at curved sections, crashes at basic roadway segments without any speed control facilities, and head-on crashes. These findings are expected to serve as a valuable reference for formulating remedial policy measures to decrease the severity of motorcycle crashes on roadways in the Seoul metropolitan area of South Korea.

## 1. Introduction

People in South Korea rarely use motorcycles as their main mode of transportation or as a mode of recreational travel. Instead, motorcycles are predominantly used for food and/or mail deliveries [1]. The majority of such motorcyclists are in their late teens and early twenties, and they are inexperienced in most cases. Motorcycle crashes account for 5% of the total traffic crashes in Korea, but death by motorcycle crash represents about 10% of the total deaths from traffic crashes [1]. Thus, evaluating the factors that affect the severity of motorcycle crashes is an important step towards developing effective policy measures and technologies to reduce the severity.

The main objective of this study is to review and evaluate the critical factors that affect motorcycle crash severity on roadways in the Seoul metropolitan area of South Korea. We also propose remedial policy measures to reduce motorcycle crash severity. The following factors related to motorcycle crashes were evaluated: crash characteristics, motorcyclist factors, characteristics of the other vehicles, and drivers involved in the crash, roadway factors, and environmental factors. These factors involve various types of measurement scales, such as nominal, ordinal, and interval scales, which makes the complexity and dimensionality of the combined dataset quite high. 

Most previous studies on motorcycle crash severity have applied linear statistical methods, in which the results are invariant under any linear transformation of the variables [2]. However, nonlinear versions based on optimal scaling techniques have been developed for use with nominally scaled variables and ordinal variables. Therefore, this study applies two nonlinear optimal scaling methods to assign numerical quantifications to the categories of each variable. This allows standard procedures to be used to obtain a solution for the quantified variables. First, to reduce the original set of variables into smaller sets of uncorrelated components [3], categorical principal components analysis (CatPCA) was applied to extract the principal components of motorcycle crashes. Second, based on the CatPCA results, a nonlinear canonical correlation analysis (NLCCA) was performed to identify the relationship between the extracted principal components and crash severity. Interpretations and discussions of the results are presented, followed by a summary of key findings and recommendations.

## 2. Literature Review

### 2.1. Motorcycle Crash Analysis

There are various approaches to identify the relationships among motorcycle crash severity, the types and occurrence rates, and causal factors. These approaches have mainly dealt with seven issues: (1) helmet use, (2) driving under the influence (DUI), (3) inexperience and recklessness, (4) speeding, (5) road geometry, (6) weather condition, and (7) vehicle type. 

Table 1 shows some examples with the methodologies used, and key findings. The methodologies include simple statistical methods such as descriptive statistics and Chi-square tests, as well as advanced statistical methods such as logit models, two-way fixed effects models, random-effects Poisson regression, ordered or proportional odds models, Cox models, and Bayesian approaches. These models are restricted in their ability to explain the joint relationship between multiple dependent variable sets and multiple independent variable sets. Moreover, the regression models (except for the Cox model) require assumptions about the underlying distribution for the observed dataset.

### 2.2. Nonlinear Optimal Scaling Method Applied to Transportation Fields

The causes of traffic crashes are generally classified into vehicle, driver (or human), and roadway factors, and each factor includes various types of variables such as numerical, ordinal, and nominal scales. Linear statistical methods, including regression, logit, discriminant analysis, and factor analysis, are limited to numerical variables and categorical variables of the dummy type. Moreover, the analysis of crash dataset needs to accommodate multiple dependent and independent variables. Canonical correlation analysis (CCA) deals with two variable sets (i.e., a dependent set and an independent set), but it only deals with scaled interval or dummy variables. This restriction can limit the usefulness of conventional CCA in investigations of traffic crash characteristics.

Nonlinear canonical correlation analysis (NLCCA) was developed to analyze the relationships among more than two sets of nominal or ordinal variables (more than one independent set and more than one dependent set). It is similar to CCA, but it quantifies (or rescales) the categories of these variables. As a result, nonlinear relationships among the variable sets can be analyzed, and the sets can be compared to an unknown compromise set defined by the object scores [25]. The results can then be interpreted easily using a graphical display. Graphical interpretation is a useful tool for exploring transportation data, which includes numerical, ordinal, and nominal types of data. Moreover, no probabilistic assumptions are made for the underlying distribution of the observed data.

In spite of such advantages, only a few studies have applied NLCCA to the transportation sector. Golob [2] used NLCCA to analyze the relationships between the activity patterns in home-based trip chains, and the characteristics of the people making the chained trips. Hensher and Golob [26] used a geometric perspective to describe the application of NLCCA in transportation research. Golob and Recker [27], and Golob et al. [28] used NLCCA to describe the relationships between crash characteristics and other crash causal factors. d’Ovidio et al. [29] applied CatPCA to choose principal components to investigate passenger satisfaction with the quality of public transportation services.

## 3. Methodology

### 3.1. Overview

**Figure 1:**
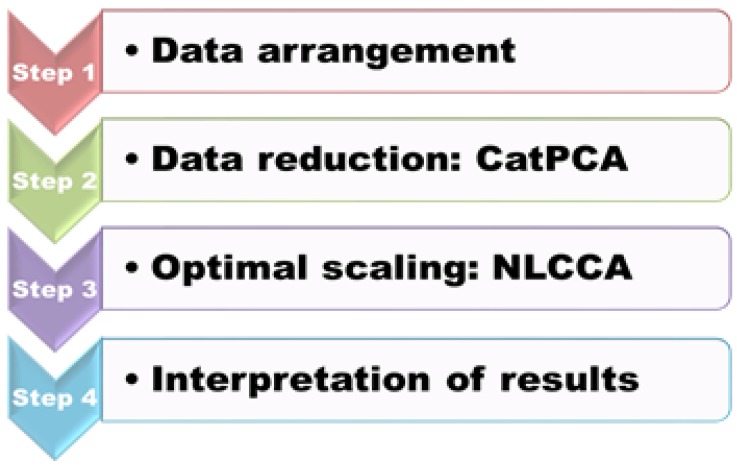
Overall framework for traffic safety analysis.

Figure 1 presents the overall process used for this study. The first step is to arrange the dataset. This step includes the selection of recorded crash data and the definition of candidate variables for the analysis. Scholars in safety analysis are often faced with a large number of variables, and they wish to reduce them to a small number of composites with as little loss of information as possible. As a second step, a state-of-the-art computer program called CatPCA is used to reduce the different types of multivariate data. CatPCA is a good tool for analyzing complicated multivariate data, which consist of nominal, ordinal, and numerical variables [30]. In the third step, categorical variables of any noninterval variables obtained from CatPCA are optimally scaled by NLCCA, which is called the OVERALS algorithm. NLCCA is also used to explore the nonlinear relationships between various crash attributes and injury severity. Finally, interpretations and discussions are based on the NLCCA results.

### 3.2. Categorical Principal Component Analysis

Although principal component analysis (PCA) is considered to be an appropriate method to perform data reduction [31], it basically suffers from two limitations: the linear relationship assumption between variables, and the variable measurement assumption to be scaled at the numeric level. These assumptions are not justified in traffic safety analyses, which are associated with nonlinear relationships among variables, as well as with nominal and ordinal variables (e.g., [32,33,34]). Particularly, if this relationship is mis-specified, the results can be heavily biased [35]. However, nonlinear PCA can incorporate nominal and ordinal variables, and handle nonlinear relationships between variables [36]. CatPCA in SPSS (Statistical Package for the Social Sciences) provides these features. 

In CatPCA, the optimal quantification process turns a qualitative nominal or ordinal variable into a quantitative numerical variable [3], and the resulting nonlinearly transformed variable can be represented as a vector in the space that is determined for the objects (or crashes in this study) [30]. Each variable is associated with a set of component loadings, one for each dimension. These loadings are correlations between the variables and the principal components, and they give coordinates for the variables to represent them as vectors in the principal component space [30]. Component loadings are comparable with factor loadings in a factor analysis. Specifically, they are equal to a Pearson correlation between the principal component, and a quantified variable if there is no missing observation.

In the graphical representation of the result (i.e., component loading plots), the graph is interpreted by using the four quadrants based on the origin (0,0). As indicated by Linting et al. [36], in the case of long vectors, the cosines of the angles between the vectors imply the correlations between the quantified variables. If associated component loadings of the variables are closely plotted within a quadrant, they are interpreted as principal components that positively affect each other. If the component loadings of a variable are within a diagonal quadrant, the variable is interpreted to be a negatively impacting factor. Such principal components increase the explanatory power of the variable, due to the increasing values of components loadings when they are far from the origin.

Given the number of dimensions in the analysis *N*, CatPCA maximizes the sum of the N largest eigenvalues of the correlation matrix between variables transformed into ordinal or nominal scales. The overall goodness-of-fit measure is the sum of the eigenvalues, which is equal to the total variance accounted for (VAF). The VAF in each dimension for each variable is equal to the component loading squared. The component loading ranges between −1 and 1, and it is the correlation between the transformed variable and a principal component in a particular dimension [30]. Although there is no guideline for acceptable component loading values, Garson [37] suggested a minimum value of ±0.30. A more detailed explanation of CatPCA and its mathematical formulations can be found in the handbook by Meulman et al. [30].

### 3.3. Nonlinear Canonical Correlation Analysis

NLCCA was first developed by a doctoral student in the Department of Data Theory at the University of Leiden in the Netherlands [38] and it was documented by Gifi [39] and van der Burg and de Leeuw [40]. The objective of NLCCA is to find what is common between sets of variables. NLCCA is a form of homogeneity analysis with restrictions. Homogeneity analysis determines transformations of the categories of variables to maximize homogeneity, and it is the basic technique in the Gifi system of descriptive nonlinear multivariate analysis. The Gifi system is characterized by the optimal scaling of categorical variables, which is implemented by using alternating least squares (ALS). The ALS algorithm is known as the OVERALS algorithm [39,41]. Unlike traditional canonical correlation analysis, no assumptions are made about the variables’ distributions, nor are the variables’ presumed to be related in a linear way [42]. Moreover, the algorithm can handle more than two sets of variables. OVERALS is available in SPSS Categories 10.0 and later versions [43].

In this study, NLCCA starts with the results of CatPCA (i.e., identified principal components). A two-dimensional analysis method can be used to analyze the relationship between crash characteristics and each influencing factor. In this method, it is estimated that there is a correlation between orthogonal factors or factors in the same quadrant. In the case of crash severity, there is a correlation among the ordinal and orthogonal variables, which necessitates a two-dimensional analysis. OVERALS in SPSS can assess commonalities between different sets of variables of the same objects. By using this tool, relationships and similarities among and within the sets of variables are analyzed. The result can be evaluated by loss and fit measures [44,45]. The interpretation of which variables are related and to what extent is based on the centroid plots.

As for the loss and fit measures, the maximum value of the loss is between 0 and *K* in case of a *K*-dimensional solution [46]. The loss can be divided over dimensions, and one minus the loss per dimension corresponds to the eigenvalue, which ranges from 0 to 1. The eigenvalue corresponds to goodness-of-fit measures, and the sum of eigenvalues is called the total fit. Therefore, the fit is maximally equal to the number of dimensions, and the loss is equal to *K* minus the total fit. Generally, no absolute value can be given as to what fit is acceptable, although fit values under 0.5 are considered to be on the brink [47].

Centroid plots can be interpreted easily by using graphical methods such as component loading plots in CatPCA. These plots show how well variables separate groups of objects with centroids at the center of gravity of the objects. To understand the relationships between variables, matching clusters of categories in centroid plots need to be identified [25]. These plots are applied to better understand individual relationships among variables. The interpretation of these plots is similar to that of the component loadings in CatPCA. That is, the distance from the origin to each variable point approximates the importance of that variable. Thus, the most effective variables in relationships among variable sets are the ones that are positioned far away from the origin. Furthermore, close variables have more similarities than variables that are far apart in a quadrant. The correlation between two variables in the centroid plot can be interpreted by using the cosine of the angle between lines from the origin to each variable: (1) positive correlation if the angle is acute, (2) negative correlation if the angle is obtuse, and (3) no correlation for right angles [48]. 

## 4. Data Arrangement

The dataset was collected from the Traffic Accident Analysis System (TAAS) of the Korea Roadway Traffic Authority). The analysis was based on the 2009 data of motorcycle crashes in the Seoul metropolitan area. A total of 4685 motorcycle crashes occurred in this area during the analysis period. Of these, only 2593 cases were associated with motorcycle-to-vehicle crashes. Motorcycle crash data from TAAS includes crash characteristics, motorcyclist characteristics, vehicle driver characteristics, roadway characteristics, and environmental characteristics, as shown in Table 2, and data were composed of five sets with respect to the different characteristics, and each variable was categorized based on the original categories in the database.

- **Set 1**: Crash characteristics were divided into the crash severity of the motorcyclist, crash type, and crash location. According to the TAAS classification, the severity of crashes was categorized into four groups: ‘death’, ‘incapacitating injury’, ‘visible injury’, and ‘complaint of pain’. Crash type was classified into five groups: ‘head-on crash’, ‘crash while parking’, ‘rear-end crash’, ‘broadside crash’, and ‘other cases’. Lastly, crash locations were categorized into ‘intersections’, ‘basic roadway segments’, and ‘crosswalks’.
- **Set 2**: Environmental characteristics have a direct influence on crashes, particularly in the case of two-wheeled motorcycles. For example, a wet roadway surface often leads to slipping while turning and stopping. This study classifies environmental factors into three categories: ‘weather’, ‘season’, and ‘roadway surface condition’.
- **Set 3**: Motorcyclist characteristics can be divided using information about the motorcyclists and their motorcycling characteristics. Information about the motorcyclist included ‘age’, ‘gender’, and ‘job’, while motorcycling characteristics included ‘DUI’, ‘crash time’, and ‘crash speed (hereafter Speed1)’. Crash speed is recorded as crash information estimated through various sources such as closed-circuit television (CCTV) data, statements of crash-involved drivers, and witness in the vicinity of a crash, and engineering estimations of post-crash trajectories and skid marks at the scene [1]. The crash time was classified into ‘AM peak time’ (7:00 to 9:00), ‘midday time’ (9:00 to 17:00), ‘PM peak time’ (17:00 to 20:00), and ‘night time’ (20:00 to 7:00).
- **Set 4**: Characteristics regarding other crashing vehicles were categorized into driver factors, such as ‘gender’, ‘age’, and ‘DUI’, as well as vehicle factors, such as the ‘size of other drivers’ vehicle’ and ‘speed’.
- **Set 5**: Roadway characteristics include geometric factors such as horizontal and vertical alignment, median types, and the width of the roadway section. The factor of horizontal alignment was classified as ‘straight’ sections and ‘curved’ sections. Similarly, the vertical alignment factor is classified as ‘uphill’, ‘downhill’, and ‘flat’ sections. The type of median is categorized into three categories: ‘none’ (18.9%), ‘painted marking’ (70.3%), and ‘barrier’ (10.9%). Finally, the directional roadway width is classified as an ordinal variable measure.

## 5. Multivariate Statistical Analysis

### 5.1. Data Reduction by Categorical Principal Component Analysis

#### 5.1.1. Selection of Principal Components

Table 3 summarizes the results of the data reduction by CatPCA. Component loading refers to the explanatory power, and the factors with high component loadings can be considered as a principal factor [3]. In this table, variables associated with the greatest component loading values are ‘weather’ and ‘road surface’, but the variables ‘season’ and ‘uphill’ have the lowest values. Based on the minimum component loading value by Garson [37], 11 principal components were selected. 

The intercorrelations of the variables were analyzed to identify multicollinearities between selected principal components. As a result, the variables ‘weather’ and ‘roadway surface condition’ had a comparatively high correlation value of 0.721, meaning that they are most likely collinear. Similarly, the correlation between the ‘night’ and ‘midday’ variables was 0.594. Because of this multicollinearity, the variables ‘weather’ and ‘midday’ were thus excluded from the analysis. Lastly, although the component loading for the variables ‘Speed2’, ‘horizontal alignment’, and ‘DUI1’ were low, they were included in the analysis due to their potential impacts on crash severity. As shown in Table 4, 11 principal components were selected as independent variables for NLCCA.

#### 5.1.2. Component Loading Plot

**Figure 2:**
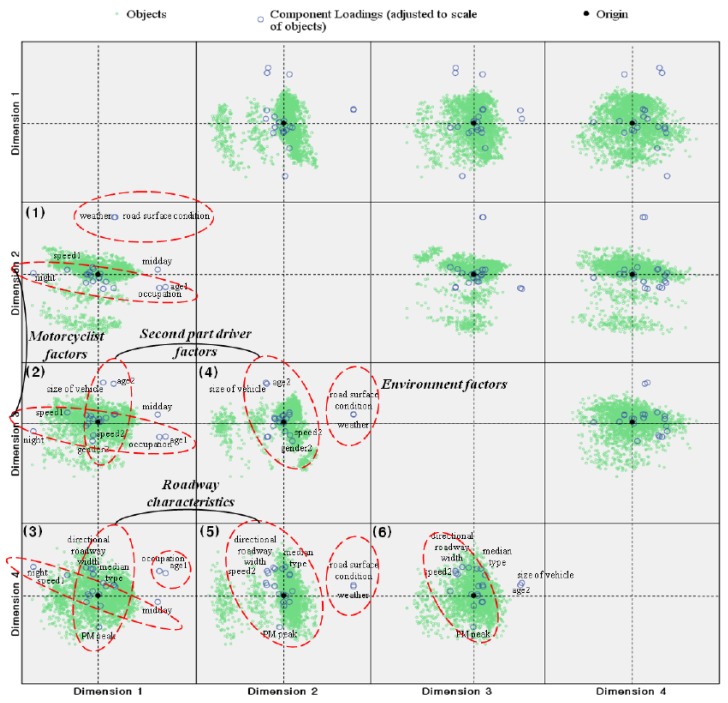
Categorical principal component analysis of variables (variable principal normalization).

Regardless of the principal component selection, the component loadings plots in Figure 2 are presented, to illustrate the relationship among the variables. This figure shows two-dimensional scatter plots organized as a 4 × 4 array. For example, the picture (Figure 2 (3)) in the lower left-hand corner of the figure is a scatter plot of objects and components loadings in the illustrated in dimension 1 and 4. In Figure 2 (1), the variables with greater component loading are ‘weather’ and ‘road surface conditions’ in the first quadrant, ‘speed1’ and ‘night’ in the second quadrant, and ‘job’ and ‘age1’ in the fourth quadrant. Since these pairs of variables are closely located, they are considered to be positively correlated. However, ‘speed1’ and ‘night’ and ‘age1’ and ‘job’ are negatively correlated because they are diagonally located with respect to the origin. Moreover, since ‘speed1’ and ‘night’ as well as ‘weather’ and ‘road surface conditions’ are perpendicularly located with respect to the origin, they are not correlated.

In Figure 2 (2), the variables associated with greater component loadings ‘age2’, ‘type of other vehicle’, and ‘midday’ are located in the north and the east areas of the first quadrant, respectively, while ‘speed1’, ‘gender2’ and ‘speed2’, and ‘age1’ and ‘job’ are located in the second, third, and fourth quadrants, respectively. This figure shows a different result from that of Figure 2 (2) in that it shows close correlations between ‘size of other vehicle’ and ‘age2’, as well as ‘gender2’ and ‘speed2’. Moreover, ‘size of other vehicle’ and ‘age2’, as well as ‘gender2’ and ‘speed2’ are negatively correlated. In Figure 2 (3), the variables associated with directional roadway width and median type are closely located in the second quadrant, and the variable ‘PM peak time’ is located in the fourth quadrant. These results indicate that directional roadway width and median type are positively correlated, and the PM peak time negatively impacts motorcycle crashes. Other results shown in Figure 2 (4), (5), and (6) can be interpreted similarly.

### 5.2. Optimal Scaling by Nonlinear Canonical Correlation Analysis

#### 5.2.1. Loss and Fit Measures

NLCCA was used to analyze the selected independent variables that affect the severity of crashes. A two-dimensional analysis method was used to analyze the relationship between the crash characteristics and each influencing factor [3]. In this method, it is estimated that there is a correlation between orthogonal factors or factors in the same quadrant. Particularly, in the case of crash severity, there is a correlation among the ordinal and orthogonal variables, which necessitates a two-dimensional analysis. By applying OVERALS in IBM SPSS, the model fit of the two-dimensional NLCCA solution is obtained as follows. An actual fit value of 0.736 was calculated for variation, which is not very high, but it is over the brink. The average loss values for dimensions 1 and 2 were 0.616 and 0.647, respectively, which indicate that almost the same portion of variance was accounted for by each of the two dimensions. Lastly, the eigenvalue (1—mean loss of each dimension), which indicates the level of relationship shown by each dimension, is not very high (0.384 for dimension 1 and 0.353 for dimension 2). The following centroid plots show a more detailed interpretation of which variables are related, and to what extent.

#### 5.2.2. Centroid Plot: Crash Characteristics and Crash Severity

**Figure 3:**
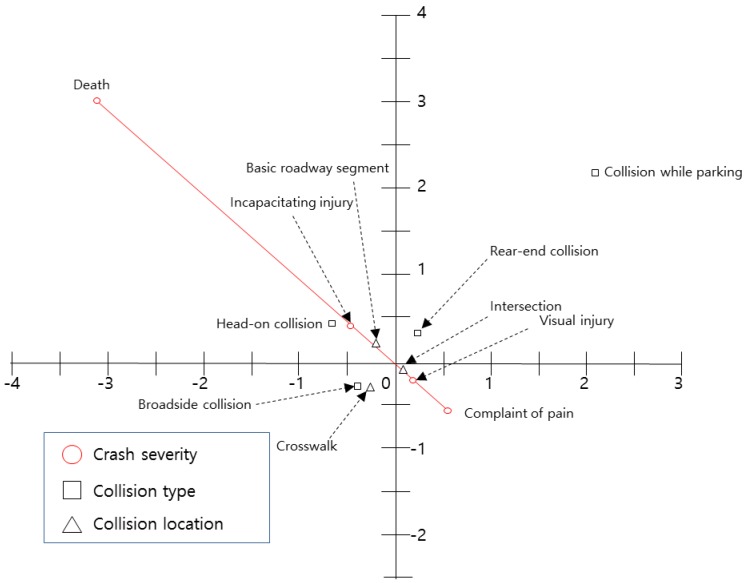
Centroid plot for crash characteristics and crash severity variables.

The relationship between crash severity and crash characteristics is shown in Figure 3. The crash severity is plotted in the second and fourth quadrants. The severity increases from the second quadrant toward the north-west, and it decreases from the fourth quadrant to the south-east. However, since there is no crash severity variable in the first and third quadrants, variables in these quadrants are not correlated with crash severity. Based on this interpretation, ‘head-on crash’ and ‘basic roadway segment’ in the second quadrant are located near ‘incapacitating injury’. However, ‘other cases’ and ‘intersections’ are close to ‘visible injury’. Thus, the results suggest that variables associated with crash severity include ‘head-on crash’, ‘basic roadway segments’, ‘other cases’, and ‘intersections’. Moreover, since the variables of ‘crashes while parking’, ‘rear-end crashes’, ‘broadside crashes’, and ‘crosswalks’ are located in the first and third quadrants, they are not correlated with crash severity.

#### 5.2.3. Centroid Plot: Motorcyclist Characteristics and Crash Severity

**Figure 4:**
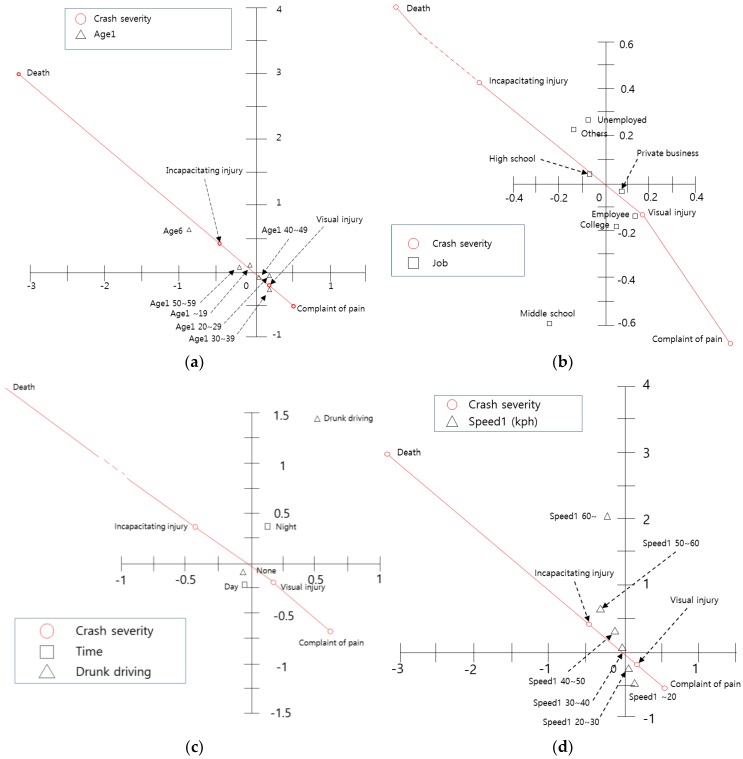
Centroid plot for motorcyclist characteristics and crash severity variables: (**a**) Centroid plot for age and crash severity variables; (**b**) Centroid plot for occupation and crash severity variables; (**c**) Centroid plot for driver time and DUI and crash severity variables; (**d**) Centroid plot for driving speed and crash severity variables.

Figure 4 shows the relationship between driver factors and crash severity. As shown in Figure 4a, the age of motorcyclists is correlated with crash severity. Particularly, since the age category ‘60s or more’ is located in the second quadrant and is plotted in the north-west area (the point representing incapacitating injury), crashes involving motorcyclists over the age of 60 may have increased the possibility of incapacitating injury or death. In addition, the variables ‘10s’ and ‘50s’ are in the second quadrant. Therefore, the results suggest that these two variables are also associated with incapacitating injury. On the other hand, ‘20s’, ‘30s’, and ‘40s’ have an influence on ‘complaint of pain’. A similar interpretation can be made for the other parts of Figure 4. 

Figure 4b shows that ‘high school students’, ‘the unemployed’, and ‘others’ have high crash severity, and that ‘private business’, ‘college students’, and ‘employees’ have less crash severity. Figure 4c shows the relationship between ‘crash time’, ‘DUI’, and ‘crash severity’, which have a great influence on crash occurrence but not crash severity. Lastly, Figure 4d shows the relationship between crash severity and speed at the time of the crash. The results show that higher speeds bring about higher crash severity, particularly speeds over 30 km/h (incapacitating injury and/or death). 

#### 5.2.4. Centroid Plot: Characteristics Regarding Other Crashing Vehicles and Crash Severity

**Figure 5:**
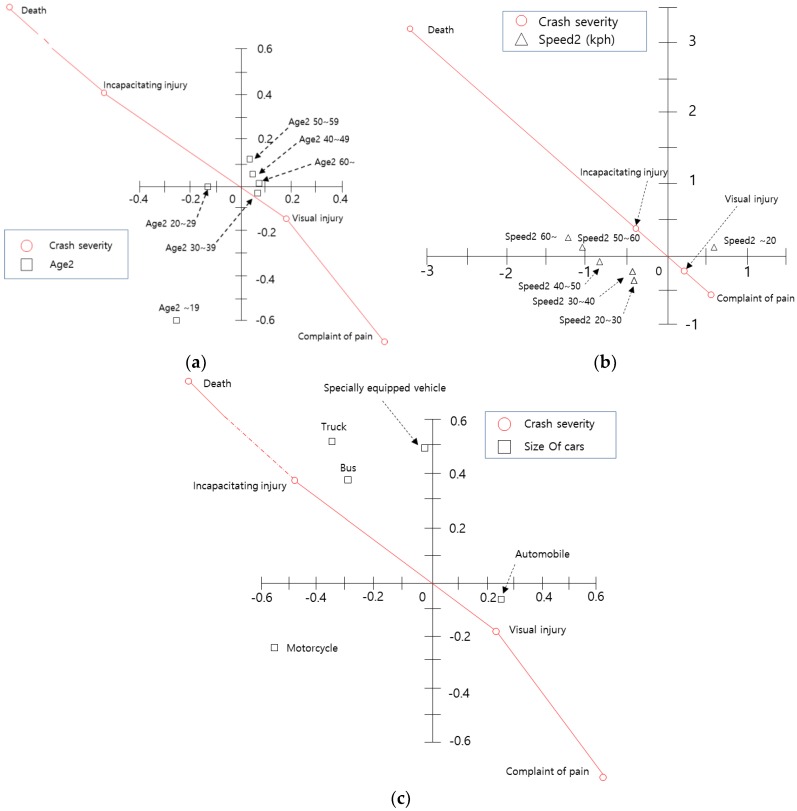
Centroid plot for characteristics regarding other vehicles and crash severity variables: (**a**) Centroid plot for age and crash severity variables; (**b**) Centroid plot for speed and crash severity variables; (**c**) Centroid plot for size of vehicle and crash severity variables.

The relationship between the characteristics regarding other crashing vehicles and crash severity is shown in Figure 5. As shown in Figure 5a, the other driver’s age has little relationship with crash severity. However, the variable ‘20s’ is located in the second quadrant, and is thus correlated with ‘incapacitating injury’. As shown in Figure 5b, ‘speed over 50 km/h’ (spd2_5 and spd2_6) has a great influence on crash severity. Finally, Figure 5b shows the relationship between other vehicle’s type and crash severity. This figure suggests that crash severity (incapacitating injury and death) increases when larger vehicles such as ‘trucks’, ‘specially equipped vehicles’, or ‘buses’ are involved in motorcycle crashes. 

#### 5.2.5. Centroid Plot: Environmental Characteristics and Crash Severity

**Figure 6:**
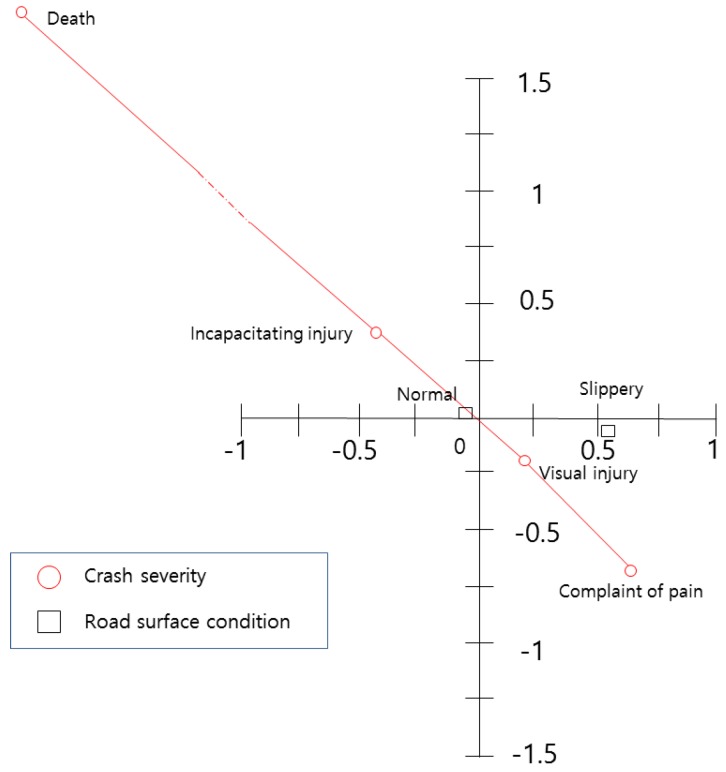
Centroid plot for roadway surface condition and crash severity variables.

The relationship between the roadway surface conditions and the crash severity is presented in Figure 6. Since the variable for normal surface conditions is located in the second quadrant, it is interpreted as a factor that increases crash severity. This result could be due to the fact that dry weather prevails all year round. However, its location is very close to the origin. Thus, this variable is not an effective one for crash severity. Slippery conditions increase the possibility of crashes due to the variable’s location, but they do not have a direct relationship with crash severity, due to the angle between the crash severity points and the variable location. 

#### 5.2.6. Centroid Plot: Roadway Characteristics and Crash Severity

**Figure 7:**
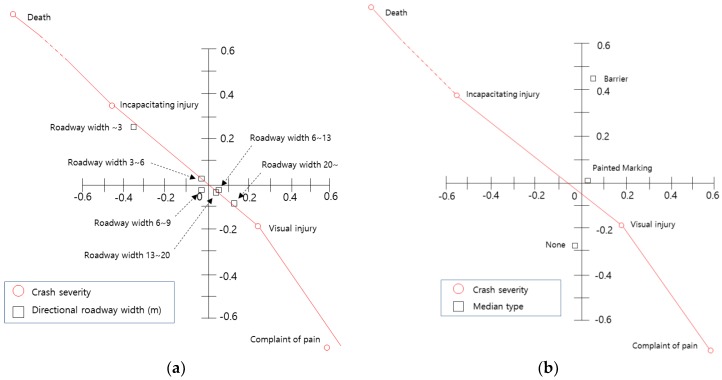

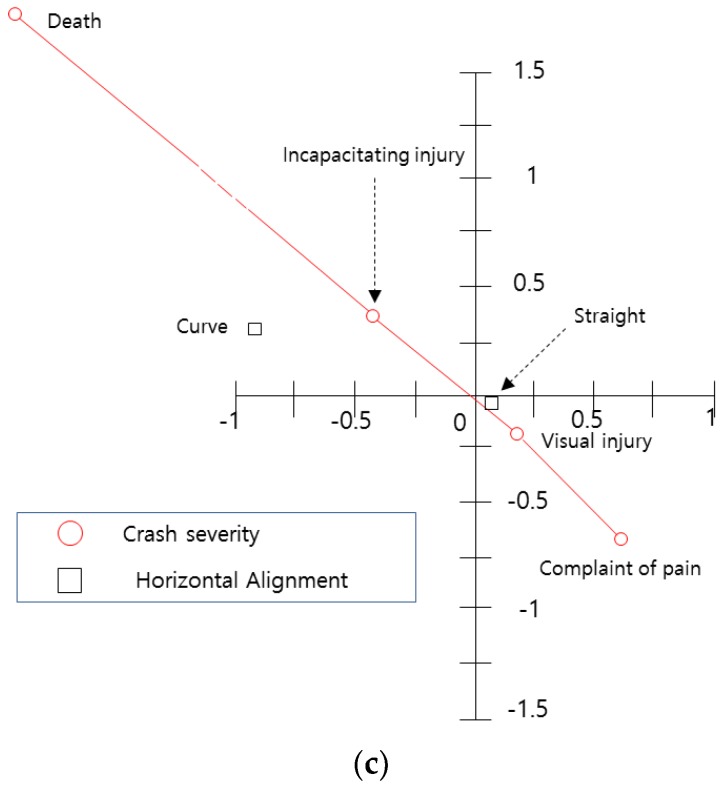
Analysis of crash severity and roadway factor variables: (**a**) Centroid plot for directional roadway width and crash severity variables; (**b**) Centroid plot for median type and crash severity variables; (**c**) Centroid plot for horizontal alignment and crash severity variables.

The relationship between crash severity, roadway facilities, and geometry is shown in Figure 7. As shown in Figure 7a, since the variables related to ‘directional roadway width of less than 6 m’ are located in the second quadrant, they are correlated with ‘incapacitating injury’. Particularly, the variable ‘directional roadway width of less than 3 m’ is more correlated with ‘incapacitating injury’ than the other variable. In other words, the narrower the roadway, the greater the crash severity is. Figure 7b shows that the presence of a median barrier has no relationship with crash severity, and Figure 7c shows that curved sections have a significant impact on crash severity.

## 6. Discussions

Severe motorcycle crashes that lead to serious injury or death incur high social cost. Thus, by identifying the factors that affect motorcycle crashes, appropriate policy measures can be instituted to minimize fatalities. The variables discussed in this study have a significant influence on motorcycle crash fatality, and they often interactively dictate the degree of crash severity. It is necessary to identify the most influential variables and to isolate them to help formulate legislative policies to reduce motorcycle crash severity. The most influential factors according to the NLCCA are shown in Table 5.

The variables in Table 5 were in the second quadrant, where both death and incapacitating injury variables are located in the centroid plots. That is, there was a significant relationship between crash severity and these variables: head-on crash, crash at basic roadway segment, younger (under 20) or older (over 50) motorcyclists, motorcycle speed over 30 km/h, speed of the other crashing vehicle over 50 km/h, crashes with heavy vehicles such as trucks, buses, and specially equipped vehicles, narrow roadways (under 6 m), and crashes at curved segments. Overall, the results are consistent with those from prior studies. Savolainen and Mannering [21] and Pai and Saleh [49] indicated that head-on crashes tended to increase crash severity. Crashes that occurred at basic roadway segments also had an effect on severe injuries such as death and incapacitating injury, which is consistent with findings by Eystace et al. [50]. 

The relationship between crash severity and other variables including age, speed, vehicle type, and roadway width and alignment have been studied previously. The trends have similar patterns to the results from this study. For example, Baker et al. [51], Braddock et al. [52], and Lardelli-Claret et al. [15] emphasized the close relationship between young motorcyclists and high crash severity, while Kraus et al. [53], Lardelli-Claret et al. [15], NHTSA [54], Lin and Kraus [55], and Albalate and Fernández-Villadangos [56] reported that older motorcyclists (e.g., over 40, over 60, or over 75) are associated with much higher crash severity.

One of the most widely studied issues in crash severity analysis is the relationship between speed and crash severity. It is natural that higher crash speed is directly related to greater crash severity. Various studies on motorcycle crash severity analysis proved this relation (e.g., [1,15,50,57,58]). Moreover, motorcycle crashes with heavy vehicles contributed to higher crash severity, which also conforms to the previous studies (e.g., [1,18,58,59]). Lastly, crash severity is also high when the roadway is less than 6 m wide and is curved. These results are consistent with findings by Hague and Chin [20], and Albalate and Fernández-Villadangos [56], who indicated that crashes on single-lane roadways have higher severities. Similarly, Hague and Chin [20], Eustace et al. [50], and Chimba and Sando [58] reported that crashes on curved sections result in higher severity. 

Based on the findings, the following remedial measures are suggested to reduce motorcycle crash severity. Firstly, a measure for maintaining reasonable speed that considers roadway conditions should be established. Currently, there are few warning signs for curved sections in the Seoul metropolitan area of South Korea. Thus, warning signs and speed limit signs should be installed at the entrances of curved sections. A plan is also needed to raise motorcyclists’ awareness of roadways that have fewer than two lanes and that are 6 m wide or less.

Secondly, there is a need for strong legal action against younger motorcyclists violating traffic laws. In addition, educational programs about the dangers of motorcycling are needed for older motorcyclists. For motorcyclists older than 50, educational programs about safe motorcycling would be very helpful and should be provided, because aging weakens cognitive control and reaction time. However, Matthews and Moran [60] reported that educational programs for younger motorcyclists did not have a significant impact on reducing reckless motorcycling habits. Guria and Leung [61] found the positive effect of enforcement and education through advertising campaigns. Therefore, such a complementary program for younger motorcyclists would be helpful for changing unsafe motorcycle habits.

## 7. Conclusions

This study identified the factors affecting the severity of motorcycle crashes occurring in the Seoul metropolitan area of South Korea during 2009. Remedial measures were suggested to reduce crash occurrence and severity. Since the collected crash data are associated with various types of measurement scales such as nominal, ordinal, and interval scales, we applied CatPCA and NLCCA. The results showed that the factors affecting the motorcycle crash severity included head-on crashes, crashes at basic roadway segment, crashes by younger (under 20) or older (over 50) motorcyclists, crashes with motorcycle speed over 30 km/h, crashes with the other crashing vehicle speed over 50 km/h, crashes with heavy vehicles, crashes on narrow roadways (under 6 m), and crashes on curved segments. These findings were all consistent with previous studies.

Since the major purpose of motorcycle trips in Seoul metropolitan area is the fast delivery of food and parcels, most motorcyclists are inexperienced, and young motorcyclists and motorcyclists are required to make fast delivery times [1]. Therefore, to develop more specified countermeasures for motorcycle safety, further analysis is required to identify the relationship between the crash severity and types of delivery for such foods and parcels. Further studies could be conducted with additional variables that can affect motorcycle crash severity. Examples include the usage of motorcycle protective clothing, congestion level, traffic volume at the crash moment, and displacement variables with respect to crashed motorcycles. Specifically, more accurate crash speed data available from vehicle state-of-the-art technologies, such as vehicle black boxes and event data recorders can be used [62,63,64]. Finally, the methods applied in this study show the relationships in relative scales based on graphical displays rather than parameter estimates. Visualizing the results from the methods can form a basis for an analysis on the interpretation of model parameters. Consequently, further studies could be done to develop parametric models based on nonlinear optimal scaling methods.

## Figures and Tables

**Table 1:** Example of recent motorcycle crash studies.

Research Issue	Author	Findings	Methodology
Helmet use	NHTSA [4]	Helmets are estimated to be 37% effective in preventing fatal injuries to motorcyclists.	Descriptive statistics
	Houston and Richardson [5]	When mandatory helmet use was reintroduced, the percentage of deaths was decreased by 21.7%.	Two-way fixed effects model
	Cook et al. [6]	Helmeted motorcyclists were significantly less likely to experience a traumatic brain injury.	Multivariate logistic regression
	Cunto and Ferreira [7]	Helmets in a motorcycle crash reduce the probability of suffering severe and fatal injuries by 9%.	Mixed ordered logit model
DUI (driving under the influence)	Kasantikul et al. [8]	DUI-related crashes are most likely to occur on weekends, at night, and en-route home.	*χ*^2^ test
	NHTSA [4]	Another factor related to motorcycle crash severity is DUI.	Descriptive statistics
	Villaveces et al. [9]		Random-effects Poisson regression
	Williams [10]		Descriptive statistics
Inexperience and recklessness	Lin et al. [11]	Inexperienced motorcyclists or those with less experience often tend to over speed, DUI, ride without a helmet, be reckless, run yellow lights, tail others, etc.	Proportional odds model
	Oluwadiya et al. [12]	Younger (less than 20 years old) and inexperienced motorcyclists are generally associated with a high degree of crash severity.	Descriptive statistics
	Lin et al. [13]		Anderson–Gill (AG) multiplicative intensity model
Speeding	NHTSA [4], Lin et al. [11,13]	High motorcycle speed was generally associated with a high degree of crash severity (twice that of car or light-truck crashes).	Proportional odds model and AG multiplicative intensity model
	Li et al., [14]		Binary logistic regression and Cox proportional hazard model
	Lardelli-Claret et al. [15]	Non-adherence to the designated speed limits contributed to crashes and the degree of severity.	Logistic regression
	Christie et al. [16]	Monitoring devices such as cameras for speeding reduced the number of crashes significantly, particularly those related to motorcycle injuries, which decreased by about 63%.	Descriptive statistics
Road geometry	Clarke et al. [17]	Young speeding motorcyclists were more prone to crashes at curves.	Descriptive statistics
	Rifaat et al. [18]	The degree of crash severity was generally higher in the loops and lollipops of streets. By contrast, however, in Calgary, Canada, crash severity was lower in parking lots, as theoretically expected.	Ordered response model
	Lin et al. [11]	Motorcycle crashes associated with stationary roadside objects exhibited higher severity compared to those involving other motorcycles or vehicles on the road.	Proportional odds model
	Daniallo and Gabler. [19]	Guardrail crash was about seven times higher in severity compared to crash types, and about 15 times higher than tree crashes	Descriptive statistics
	Hague and Chin [20]	Motorcycle crashes are more likely to occur on single-lane roads, curbs, and on the median lanes of multi-lane roads, with a potential high degree of severity.	Mixed logit model
Weather condition	Savolainen and Mannering [21]	Visibility is poorer on horizontal curvatures, vertical curvatures, or in darkness	Unordered probability model (multinomial logit model)
	Shipp et al. [22]	Riding in poor weather conditions has an influence on fatality.	Logistic regression
	Cheng et al. [23]	Motorcycle crashes are less likely to occur during the rainfall, and the higher the air temperature, the less the probability of a fatal crash.	Full Bayesian hierarchical approach
Vehicle type	Blackman and Haworth [24]	Motorcycle crashes are more severe than moped and scooter crashes.	Ordered probit model

**Table 2:** Definition of variable sets.

Set	Variable		Number of Crashes (%)	Code	Measure
Crash characteristics	Crash severity	Complaint of pain Visible injury Incapacitating injury Death	382 (14.7%) 1297 (50.0%) 868 (33.5%) 46 (1.8%)	1 2 3 4	Ordinal
	Crash type	Head-on crash Crash while parking Rear-end crash Broadside crash Other cases	162 (6.2%) 162 (6.2%) 360 (13.9%) 1713 (66.1%) 196 (7.6%)	1 2 3 4 5	Nominal
	Crash location	Intersection Basic roadway segment Crosswalk	1466 (56.5%) 1009 (38.9%) 118 (4.6%)	1 2 3	Nominal
Environmental characteristics	Weather	Clear Others	2320 (89.5%) 273 (10.5%)	0 1	Nominal
	Roadway Surface Condition	Normal Slippery	2332 (89.9%) 261 (10.1%)	0 1	Nominal
	Season	Spring Summer Fall Winter	593 (22.9%) 806 (31.1%) 764 (29.5%) 430 (16.5%)	1 2 3 4	Nominal
Motorcyclist characteristics	Gender1	Male Female	2534 (97.7%) 59 (2.3%)	1 0	Nominal
	Age1	–19 20–29 30–39 40–49 50–59 60–	814 (31.4%) 656 (25.3%) 357 (13.8%) 384 (14.8%) 180 (6.9%) 202 (7.8%)	1 2 3 4 5 6	Ordinal
	Job	High school student College student Unemployed Private business Middle school student Employee Others	423 (16.3%) 123 (4.7%) 317 (12.2%) 370 (14.3%) 41 (1.6%) 822 (31.7%) 497 (19.2%)	1 2 3 4 5 6 7	Nominal
	DUI1	Drunk driving None	179 (6.9%) 2414 (93.1%)	1 0	
	Crash time	AM peak time Midday time PM peak time Night time	135 (5.2%) 975 (37.6%) 525 (20.3%) 958 (37.0%)	1 2 3 4	
	Speed1 (km/h)	–20 20–30 30–40 40–50 50–60 60–	765 (29.5%) 645 (24.9%) 557 (21.5%) 311 (12.0%) 215 (8.3%) 100 (3.9%)	1 2 3 4 5 6	Ordinal
Characteristics regarding other crashing vehicles	Gender2	Male Female	2212 (85.3%) 381 (14.7%)	1 0	Nominal
	Age2	–19 20–29 30–39 40–49 50–59 60–	121 (4.7%) 403 (15.5%) 594 (22.9%) 752 (29.0%) 519 (20.0%) 204 (7.9%)	1 2 3 4 5 6	Ordinal
	DUI2	Drunk driving None	25 (1.0%) 2568 (99.0%)	1 0	Nominal
	Size of other vehicle	Motorcycle Automobile Bus Truck Specially equipped vehicle ^1^	491 (18.9%) 1616 (62.3%) 182 (7.0%) 187 (7.2%) 117 (4.5%)	1 2 3 4 5	Nominal
	Speed2 (km/h)	–20 20–30 30–40 40–50 50–60 60–	1353 (52.2%) 497 (19.2%) 302 (11.6%) 208 (8.0%) 158 (6.1%) 75 (2.9%)	1 2 3 4 5 6	Ordinal
Roadway characteristics	Horizontal alignment	Curved Straight	131 (5.2%) 2462 (94.9%)	1 0	Nominal
	Vertical alignment	Uphill Downhill Flat	189 (7.3%) 229 (8.8%) 2175 (83.8%)	3 2 1	
	Median type	None Painted marking Barrier	489 (18.9%) 1822 (70.3%) 282 (10.9%)	1 2 3	
	Directional roadway width (meters)	–3 3–6 6–9 9–13 13–20 20–	226 (8.7%) 646 (24.9%) 390 (15.0%) 377 (14.5%) 454 (17.5%) 500 (19.3%)	1 2 3 4 5 6	Ordinal

^1^ This vehicle type includes trucks with trailers, oversize load trucks, and tow trucks towing car.

**Table 3:** Component loadings variable in CatPCA.

Factors	Variables	Dimension			
		1	2	3	4
Motorcyclist characteristics	Gender1	−0.107			
	Age1	0.733			
	Occupation1	0.666			
	DUI1	−0.130			
	Crash speed	−0.332			
	Crash time Night AM peak Midday PM peak	−0.700 0.087 0.647 0.012			
Environment characteristics	Weather		0.866		
	Roadway surface condition		0.867		
	Season		−0.002		
Characteristics regarding other crashing vehicles	Gender2			−0.030	
	Age2			0.724	
	Size of other drivers’ vehicle			0.747	
	Speed2			−0.248	
Roadway characteristics	Directional roadway width				0.404
	Horizontal alignment				0.020
	Downhill				−0.156
	Uphill				−0.004
	Median type				0.404

**Table 4:** Selected principal components.

Factor	Principal Component
Motorcyclist characteristics	age1, job, speed1, night, DUI1
Environment characteristics	roadway surface condition
Characteristics regarding other crashing vehicles	age2, size of other drivers’ vehicle, speed2
Roadway characteristics	directional roadway width, median type, horizontal alignment

**Table 5:** Variables of affecting crash severity increase.

Factor	Variables
Crash characteristics	head-on crash, basic roadway segment
Motorcyclist characteristics	age1 (~19, 50~), speed1 (30~)
Environment characteristics	—
Characteristics regarding other crashing vehicles	speed2 (50~), size of other drivers’ vehicle (truck, bus, specially equipped vehicle)
Roadway characteristics	directional roadway width (~6), horizontal alignment (curve)
